# Dr. George Bingham Fowler

**Published:** 1907-03

**Authors:** 


DR. GEORGE BINGHAM FOWLER, of New York, died at his home in that city March 6, 1907, from hemorrhage of the stomach, in the sixtieth year of his age. Dr. Fowler graduated at the College of Physicians and Surgeons, now the medical department of Columbia University, in 1871, and soon afterward became assistant to Professor John C. Dalton in the department of physiology, where he remained for eight years. He was Health Commissioner of New York during the Strong administration and since 1886 had been professor of clinical medicine at the Post-Graduate Medical School and Hospital. Dr. Fowler was a man of versatile talents, distinguished as a teacher and writer and was a citizen with high ideals.

